# Evaluating the Hospital Preparedness for Emergencies using WHO Hospital Safety Index Tool

**DOI:** 10.12669/pjms.41.3.10875

**Published:** 2025-03

**Authors:** Ume Sughra, Asmaa Riaz, Marriam Suleman, Muhammad Rafay Imran

**Affiliations:** 1 Ume Sughra, MCPS-HPE, FRCP, FCPS, MPH, MBBS Al-Shifa Research Centre, Al-Shifa Trust Eye Hospital, Rawalpindi, Pakistan; 2 Asmaa Riaz, MSPH, BDS Al-Shifa Research Centre, Al-Shifa Trust Eye Hospital, Rawalpindi, Pakistan; 3 Marriam Suleman, MSPH, MBBS Al-Shifa Research Centre, Al-Shifa Trust Eye Hospital, Rawalpindi, Pakistan; 4 Muhammad Rafay Imran, Higher Secondary (A Level) The Science School Al-Shifa Research Centre, Al-Shifa Trust Eye Hospital, Rawalpindi, Pakistan

**Keywords:** CBRN, Disaster, Emergency, Safety, Response, Preparedness

## Abstract

**Objective:**

CBRN (an acronym for Chemical, Biological, Radiological, and Nuclear) events are large scale disasters that differ from man- made disasters in that the involved agents additionally cause contamination and specific health hazards. As an institution, hospitals need a specific level of preparedness to enable an effective response to CBRN emergencies. The aim of this study was to assess the presence and placement of a proper hospital response plan to CBRN events.

**Methods:**

It was an observational cross-sectional study conducted from October 2021 to December 2021 in two public tertiary level healthcare facilities selected through purposive sampling technique. Data was collected using WHO Hospital Safety Index Tool which is a checklist consisting of 151 items divided into four sections or modules. To calculate the hospitals safety index Model-1 was used.

**Results:**

The sum of the weighted results of the three modules for both hospitals were in category A (0.66 – 1), the results depicted that it is likely that the hospitals will function in emergencies and disasters.

**Conclusion:**

Both hospitals gave satisfactory results when evaluated on the basis of WHO Hospital Safety Index Tool for emergency preparedness.

## INTRODUCTION

The CBRN events are large scale disasters that differ from man- made disasters in that the involved agents additionally cause contamination and specific health hazards that depends on the agent involved, incident conditions - weather, terrain, time – and exposures to life, systems, and the environment. The varied health effects of the agent used may include chemical/radiation burns, heat burns, injuries, infection, poisoning, disabilities, etc.Click or tap here to enter text.^1^,^2^ CBRN events involve unique hazards and require specific education and training for EMS providers.^3^

Pakistan is a low middle-income country situated in South Asia. Its position and geo-political challenges since the last two decades have made it more prone and vulnerable to CBRN events. Limited studies have been done in Pakistan regarding disaster preparedness. A study done in Karachi, in two public tertiary care hospitals in 2007, revealed that 46.2% participants had no idea about triaging in a mass disaster. Shabbir et al showed that amongst 156 respondents from nursing staff in Lahore, 60.9% knew what disaster preparedness is but 75.6% had never been part of any disaster management plan.^4^,^5^ Registered nurses of two hospitals in Lahore were found to have insufficient current knowledge of disaster preparedness and perhaps left too much to free initiatives. Despite this they were aware of their education and training needs and were willing to take continuing education programs to pre-pare themselves for disasters.^6^,^7^

The purpose of this study was to develop a standard national tool to evaluate hospital preparedness involving Emergency Departments (ED) as well as Emergency Medical Service (EMS) providers’ readiness for CBRN events in Pakistan. This would also help to develop a national strategy/Approach to enhance Hospital-And Emergency Medical Service (EMS) provider preparedness for CBRN events involving the minimum requirements to meet IHR-CBRN preparedness recommendations in hospitals of Islamabad Capital Territory and Rawalpindi by using WHO Hospital Safety Index evaluation forms. The objectives were to evaluate the preparedness of public health facilities for managing Chemical, Biological, Radiological and Nuclear (CBRN) events to meet the minimum IHR requirements in Islamabad Capital Territory and Rawalpindi, Pakistan.

## METHOD

An observational cross-sectional study conducted from October 2021 to December 2021 within the two public hospitals of Islamabad and Rawalpindi. The study population were the health care workers, Emergency medical professionals including doctors, nurses and paramedical staff and the hospital/health facility. The sample comprised of two public tertiary level healthcare facilities selected through purposive sampling technique.

### Ethical Approval:

Data was collected after obtaining the approval from Ethical Review Committee (Ref: 80/AST-21, Date: 14/June/2021) and later from ERRB and IERC of both hospitals.

Data was collected by using WHO Hospital Safety Index Tool developed in 2007-2008 by a group of experts within the Disaster Mitigation Advisory Group (DIMAG) of the Pan American Health Organization (PAHO). It is used for assessing operational and institutional capacity of a hospital during and after an emergency and its preparedness in managing CBRN events. It is a checklist consisting of 151 items, each reflecting an aspect of hospital safety, and weighted according to the degree of its influence on the facility’s safety. The checklist is divided into four sections or modules. To calculate the hospitals safety index Model 1 was used.^8^ Data analysis was done by entering the results from the checklist into the hospital safety index Calculator that has a series of formulas that assign specific values to each item. The calculations were based on how the evaluators rated each item and the relative importance of that item in each module and the overall safety of the hospital in case of emergencies or disasters. Depending on the safety index score, the hospitals assessed were assigned to one of three groups.

## RESULTS

Structural safety was evaluated on information obtained by initial review of technical documents and information related to the construction of the hospitals. The building of both Hospitals showed normal aging. Hazard protection measures such as mobile fire extinguishers and emergency exits were present in Hospital-A whereas no structural features were installed in Hospital-B.

### Nonstructural Safety:

Hospital-A had one main entrance and Hospital-B had two. Some measures of security protection like CCTV and security guards were available in both hospitals. Existing electrical system of Hospital-A was in good working condition with alternate source of electricity present covering more than 70% of demand in critical areas. Hospital-B also had a satisfactory system with storage areas well secured and covering the hospital demand.

### Emergency and Disaster Management:

It was observed that Disaster committee in Hospital-A existed with four departments but was not functioning effectively whereas in Hospital-B proper disaster committee and plan was in place for each member of disaster management committee.

### Weighted contribution of each to Module:

Two hospitals (Hospital-A and Hospital-B) were assessed and weighted contribution of all the items was calculated for each module. Each item was valued with a certain level of safety: “Low”, “Average” or “High”. The assessment scores (high, average, or low) for each individual item were then processed using the Safety Index Calculator (Table-I).

**Table-I:** Weighted Contribution of Parameters to each Module.

MODULE 2. Elements related to the structural safety of the hospital							
	Weighted contribution to Module (%)						
	HOSPITAL-A			HOSPITAL-B			
	Low	Average	High	Low	Average	High	Total
2.1 Prior events and hazards affecting building safety	0.00	16.67	8.33	0.00	12.50	12.50	25.00
2.2 Building integrity	0.00	33.00	42.00	5.00	55.00	15.00	75.00
Total	0.00	49.67	50.33	5.00	67.50	27.50	100.00
*MODULE 3: Elements related to the non-structural safety of the hospital*							
3.1 Architectural safety	0.00	2.41	20.59	0.00	19.71	3.29	23.00
3.2 Infrastructure protection, access and physical security	0.00	7.50	2.50	5.00	2.50	2.50	10.00
3.3 Critical systems	15.52	28.81	5.67	14.17	27.74	10.10	50.00
3.3.1 Electrical systems	0.00	6.70	3.30	0.00	8.00	2.00	10.00
3.3.2 Telecommunication systems	1.36	3.10	0.54	0.00	3.57	1.43	5.00
3.3.3 Water supply system	0.00	8.80	1.20	1.67	6.67	1.67	10.00
3.3.4 Fire protection system	5.63	1.88	0.00	6.00	1.50	0.00	7.50
3.3.5 Waste management systems	0.25	1.88	0.38	0.00	0.00	2.50	2.50
3.3.6 Fuel storage systems	4.29	0.71	0.00	2.50	2.50	0.00	5.00
3.3.7 Medical gases systems	0.00	4.75	0.25	2.50	2.50	2.00	5.00
3.3.8 Heating, ventilation, and air-conditioning (HVAC) systems	4.00	1.00	0.00	1.50	3.00	0.50	5.00
3.4 Equipment and supplies	0.00	16.15	0.85	0.85	12.88	3.27	17.00
3.4.1 Office and storeroom furnishings and equipment (fixed and movable)	0.00	0.85	0.85	0.85	0.00	0.85	1.70
3.4.2 Medical and laboratory equipment and supplies used for diagnosis and treatment	0.00	15.30	0.00	0.00	12.88	2.42	15.30
Total	15.52	54.87	29.61	20.02	62.83	19.15	100.00
*MODULE 4. Emergency and Disaster Management*							
4.1 Coordination of emergency and disaster management activities	5.55	9.45	0.00	0.00	0.00	15.00	15.00
4.2 Hospital emergency and disaster response planning	11.34	6.66	0.00	0.00	0.00	18.00	18.00
4.3 Communication and information management	0.00	7.00	0.00	0.00	1.75	5.25	7.00
4.4 Human resources	0.00	6.00	14.00	0.00	12.00	8.00	20.00
4.5 Logistics and finance	0.00	6.00	2.00	0.00	0.00	8.00	8.00
4.6 Patient care and support services	3.75	13.75	7.50	2.78	5.56	16.67	25.00
4.7 Evacuation, decontamination and security	1.40	5.60	0.00	1.40	1.40	4.20	7.00
Total	22.04	54.46	23.50	4.18	20.71	75.12	100.00

### Module-Specific Index:

Modules were evaluated individually to generate a module-specific safety index for both hospitals. For structural safety both hospitals had highest portion of safety level was “Average” indicating that both hospitals are likely to function. Highest weightage in Nonstructural Safety Module was also “Average” for both Hospitals. For Module 4, Hospital B had highest portion of safety level as “High” indicating that it is highly likely to function in an emergency or disaster situation, whereas for Hospital A safety level was “Average” (Fig.2). Using the WHO scoring calculator, a Crude safety index, Safety index and vulnerability index of each module was calculated (Table-II).

**Fig.1:**
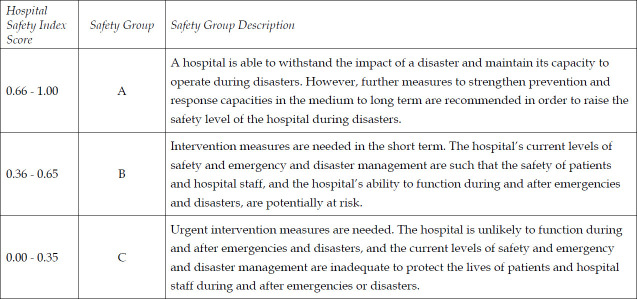
Hospital Safety index.

**Fig.2:**
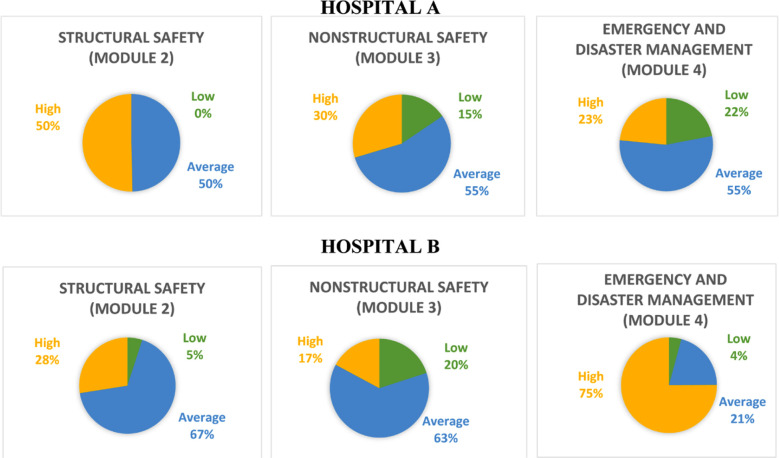
Module Specific Index of Hospitals. Both hospitals were classified as “A” based on the overall hospital safety index score, indicating that it is likely that the hospital will function in emergencies and disasters.

**Table-II:** Module Specific Indices of Hospital.

	MODULE 2		MODULE 3		MODULE 4	
	Hospital-A	Hospital-B	Hospital-A		Hospital-B	Hospital-A	Hospital-B	
*Horizontal Weight*								
Unlikely to function	0.00	1.25	3.88		5.01	5.51	1.045	
Likely to function	24.835	33.75	27.435		31.42	27.32	10.355	
Highly likely to function	50.33	27.50	29.61		17.15	23.50	75.12	
Vertical Weight	45.00	45.00	24.00		21.30	15.60	15.60	
Crude Safety Index of Module	37.67	30.41	24.42		23.68	21.66	58.62	
Range of Module	50.33	32.50	25.73		26.41	21.81	74.08	
Safety Index of Module	0.90	0.90	0.80		0.71	0.78	0.78	
Vulnerability Index of Module	-0.09	-0.09	0.20		-0.25	0.22	0.22	
Health Facility Status of Module	a	a	a		a	a	a	

### Overall Safety and Vulnerability Index:

**Fig.3:**
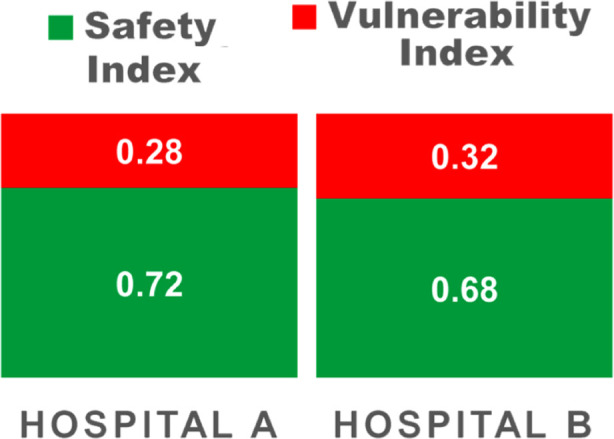
Overall Safety and Vulnerability Index of Hospitals.

To calculate overall safety index and vulnerability index, the vertical weights of each module were used. Hospital-A had a Safety probability of 72% and Hospital-B had a 32% vulnerability (Fig3).

## DISCUSSION

It is crucial for hospitals to remain operational during and after major events and disasters therefore hospital readiness and resiliency is important for a disaster-prone country like Pakistan. The Sendai Framework for Disaster Risk Reduction 2015-2030 also emphasizes on disaster prevention and risk reduction measures at critical facilities, especially in hospitals.^9^ According to hospital safety index guide (WHO, 2015), module 2 addresses the structural elements.^8^ According to Facility Guidelines Institute (FGI), Hospital-Buildings should be such that they are resilient to any disaster and should remain operational during crisis.^10^ The assessment scores for each individual item in this module was high “a” in Hospital-A and average “b” in Hospital-B indicating that the structural elements of both hospitals were likely to remain functional in an emergency.

Nonstructural elements were assessed with scores for each individual item in this module were found to be average “b” in both hospitals. According to National Fire Protection Association (NFPA 99), the hospital must have a proper fire protection system within the hospitals for the safety of patients and staff.^11^ Both hospitals lacked a proper fire protection system. The scores for each individual item in Module 4 suggested that Emergency and Disaster Management still needed improvement to function effectively in both hospitals. An increase in human resource, their training, documentation of the response plan and a designated Emergency Operations Centre (EOC) center is needed especially in Hospital-B. This was suggested in another study where it was reported that despite a high level of motivation and dedication of medical responders in Pakistan, they were not confident about handling disasters of such a high magnitude as they were not trained for such events.^12^

In another study done in Saudi-Arabia weaknesses were found in the education, training, and monitoring of the hospital staff in their preparedness for a disaster emergency.^13^ Based on the score of module-specific indices, all modules of both hospitals were classified as “a” (corresponding to a score from 0.66 to 1). The overall hospital safety index score, the status of both the hospitals was classified as “A” indicating that it is likely that the hospital will function in emergencies and disasters. This was in contrast to the study in Indonesia where all the hospitals assessed were in category “B”.^14^ In a comparative study between Iran and Sweden, the hospitals assessed in Sweden were in category “A” while those in Iran were all categorized as level “B” with respect to functional capacity.^15^

### Strength:

The study helped in identifying the hospital’s emergency preparedness for managing CBRN events in Pakistan to meet the International Health Regulations using WHO Safety Index tool.

### Limitations:

For future Private sector hospitals should also be assessed for their readiness.

### Recommendations:

This study recommends the availability of trained Human Resource and a proper Emergency recovery plan with every hospital.

## CONCLUSION

Both hospitals gave satisfactory results when evaluated on the basis of WHO Hospital Safety Index Tool for emergency preparedness.

### Author’s Contribution:

**US:** Conceived, protocol design, literature search, data collection, statistical analysis, interpretation of data, drafting of manuscript and is responsible for the accuracy and integrity of research.

**AR:** Statistical analysis, Interpretation of data, Drafting of manuscript and final approval of manuscript.

**MS:** Literature search, Data collection, Statistical Analysis and final approval of manuscript.

**MRI:** Data Collection, Manuscript Editing and Proof reading.
